# Search for Modified DNA Sites with the Human Methyl-CpG-Binding Enzyme MBD4

**Published:** 2017

**Authors:** D. A. Yakovlev, A. A. Kuznetsova, O. S. Fedorova, N. A. Kuznetsov

**Affiliations:** Institute of Chemical Biology and Fundamental Medicine, Siberian Branch of the Russian Academy of Sciences, Lavrentieva ave. 8, Novosibirsk, 630090, Russia; Department of Natural Sciences, Novosibirsk State University, Pirogova str. 2, Novosibirsk, 630090 , Russia

**Keywords:** MBD4, DNA demethylation, DNA repair, pre-steady-state kinetics, conformational dynamics

## Abstract

The MBD4 enzyme initiates the process of DNA demethylation by the excision of modified DNA bases, resulting in the formation of apurinic/apyrimidinic sites. MBD4 contains a methyl-CpG-binding domain which provides the localization of the enzyme at the CpG sites, and a DNA glycosylase domain that is responsible for the catalytic activity. The aim of this work was to clarify the mechanisms of specific site recognition and formation of catalytically active complexes between model DNA substrates and the catalytic N-glycosylase domain MBD4cat. The conformational changes in MBD4cat and DNA substrates during their interaction were recorded in real time by stopped-flow detection of the fluorescence of tryptophan residues in the enzyme and fluorophores in DNA. A kinetic scheme of MBD4cat interaction with DNA was proposed, and the rate constants for the formation and decomposition of transient reaction intermediates were calculated. Using DNA substrates of different lengths, the formation of the catalytically active complex was shown to follow the primary DNA binding step which is responsible for the search and recognition of the modified base. The results reveal that in the primary complex of MBD4cat with DNA containing modified nucleotides, local melting and bending of the DNA strand occur. On the next step, when the catalytically competent conformation of the enzyme-substrate complex is formed, the modified nucleotide is everted from the double DNA helix into the active center and the void in the helix is filled by the enzyme’s amino acids.

## INTRODUCTION

The processes of DNA methylation and demethylation are the basis for the epigenetic regulation of gene expression, which plays a major role in cellular differentiation, genome imprinting, carcinogenesis, and many age-related changes in organisms. It is known that different enzyme systems are involved in DNA demethylation: DNA methyltransferases, dioxygenases, and DNA glycosylases [1, 2]. DNA glycosylases initiate the process of demethylation through the excision of the methylated DNA base, and hence, other enzymes, such as AP-endonucleases (APE1), DNA polymerases, and DNA ligases, are necessary to restore the original nucleotide [3-5].

DNA glycosylase MBD4 [EC 3.2.2.-] contains two domains: a methyl-CpG-binding domain and a DNA gly cosylase domain. The catalytic N-glycosylase domain MBD4cat consists of 138 amino acid residues (residues 437–574) that form nine α-helices to create a globular structure and the active site pocket [6]. Based on the MBD4cat structure, the enzyme belongs to the helix-hairpin-helix family (HhH) and is so named due to the α7-loop-α8 motif, which is specific to different proteins of this family.

The main substrate of MBD4 is DNA that contains non-complementary G/T or G/U pairs [2]. MBD4 is also active on 5-hydroxymethyluracil (5-hmU) [7]. It is believed that 5-hmU is formed as an intermediate in a multistep pathway of active demethylation in which 5-meC is hydroxylated by TET-dioxygenases, with the formation of 5-hydroxymethylcytosine (5-hmC). Thereafter, 5-hmC is deaminated by deaminase AID to form 5-hmU and is removed with MBD4 during the base excision repair pathway [8]. MBD4 is also active on several halogenated substrates: 5-ClU and 5-BrU produced during inflammatory processes and 5-FU, which can appear during chemotherapy [9]. Moreover, the enzyme is active towards 3,N4-ethenocytosine (εC), which is formed during lipid peroxidation and vinyl chloride metabolism [10].

**Fig. 1:**
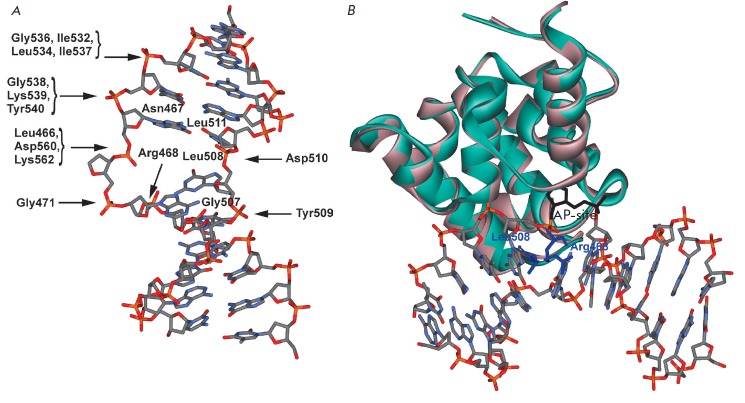
(A) Schematic representation of specific contacts in the complex between MBD4cat and DNA [12]. (B) Overall structures of free MBD4^cat^ (pink, PDB ID 4E9E, [11]) and MBD4^cat^ associated with damaged DNA (green, PDB ID 4DK9, [12]). AP-site (black) is flipped out of the double helix and inserted into the active site, Arg468 and Leu508 (blue) intercalate the DNA.

The structures of free MBD4cat and MBD4cat in complexes with DNA duplexes containing 5-hmU/G, T/G, and AP/G base pairs have been identified [11, 12]. As shown in *Fig. 1A*, in a complex with reaction products, the enzyme interacts preferably with five nucleotides of the damaged DNA strand. The formation of the enzyme-substrate complex does not lead to significant conformational changes compared to a free protein (*Fig. 1B*). Meanwhile, DNA duplexes, in complex with MBD4cat, are bent in the region of the modified nucleotide, the nucleotide is everted from the double helix, and the nitrogenous base is inserted to the active center. MBD4cat forms direct contacts with five phosphate groups located on the 5’ and 3’-side of the everted nucleotide [12]. Two amino acid residues, Arg468 and Leu508, intercalate DNA through the minor groove and fill the void in the duplex, which is formed after eversion of the modified nucleotide. It has been suggested that the hydrolysis of the N-glycoside bond occurs through a mechanism of nucleophilic substitution [7]. The C1'-atom is subjected to a nucleophile attack from either a water molecule coordinated in the active site of the enzyme or the carboxyl group of Asp560 [7, 12].

The pre-steady-state kinetics of product formation during the N-glycosylase reaction of the G/T-containing DNA substrate catalyzed by MBD4 in single-turnover conditions has been studied previously in [13] for the time range 15 s – 10 h. It has been shown that the reaction curve is biphasic with a rapid initial burst of product formation, followed by a slower phase; this is due to tight binding of the enzyme to the AP site reaction product.

The aim of this work was to study the mechanisms of specific site recognition by the enzyme in the substrate and formation of a catalytically competent state. The conformational dynamics of MBD4cat and model DNA substrates at short time points ranging from 2 ms to 200 s under conditions corresponding to or close to single-turnover of the enzyme has been studied. The changes in the protein conformation were recorded from changes in the tryptophan (Trp) fluorescence intensity. The changes in DNA conformation were studied based on the 2-aminopurine (aPu) fluorescence intensity or from the efficiency of the fluorescence resonance energy transfer (FRET) using the FAM/BHQ1 dye pair. The substrates were duplexes containing an U/G pair. The product analogue was duplexes containing an uncleavable analogue of the AP-site – (3-hydroxytetrahydrofuran-2-yl) methyl phosphate residue (F-site). The influence of the duplex length on enzyme binding to DNA and its search for modified bases was studied using substrates of different lengths: 12, 17, and 28 base pairs (bp). Based on the findings, we determined the kinetic mechanism of conformational rearrangements of DNA glycosylase MBD4cat and DNA substrates containing modified nucleotides during their interaction.

## MATERIALS AND METHODS

These reagents manufactured by Sigma-Aldrich (USA) were used: acrylamide, N,N’- methylenebisacrylamide, dithiothreitol, urea, EDTA, acetonitrile, glycerol, tris- (hydroxymethyl)-aminomethane, and domestic reagents of extra-pure grade. All solutions were prepared in double-distilled water.

**DNA-substrates**

**Table 1:** Sequences of oligodeoxynucleotides and structure of the modified residues.

Short name	Sequence
U_12_-substrate	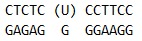
F_12_-ligand	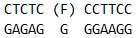
FaPu_12_-ligand	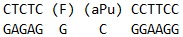
U_17_-substrate	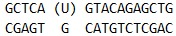
UaPu_17_-substrate	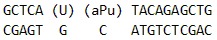
FAM-U-BHQ1-substrate	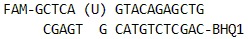
U_28_-substrate	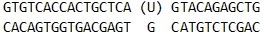

Oligodeoxyribonucleotides were synthesized at the Laboratory of Bionanotechnology, Institute of Chemical Biology and Fundamental Medicine, Russian Academy of Sciences, using an ASM-800 automated DNA/ RNA-synthesizer (Biosset, Novosibirsk, Russia) with commercial amidophosphite derivatives of nucleosides and CPG carriers (GlenResearch, USA). Native and modified oligodeoxyribonucleotides were purified with HPLC using an Agilent 1200 chromatograph (USA) and a Zorbax SB-C18 column (5 μm), 4.6 × 150 mm, with a linear gradient of acetonitrile (0 → 50%) in the presence of 20 mM triethylammonium acetate, pH 7, for 30 min at a flow rate of 2 mL/min. Fractions containing oligodeoxyribonucleotides were dried in vacuum, dissolved in water, and precipitated with 2% LiClO_4_ in acetone. After washing with pure acetone and drying, the oligodeoxyribonucleotide precipitate was dissolved in water and stored at –20°C until usage. The homogeneity of purified oligodeoxynucleotides was evaluated by denaturing gel-electrophoresis (20% polyacrylamide gel, 8 M urea, 0.1 M tris-borate buffer, pH 8.3). The oligodeoxyribonucleotides were visualized with the Stains-All dye (Sigma, USA). The substrates and ligands of the enzyme were the 12-, 17- and 28-bp oligodeoxyribonucleotide duplexes presented in *Table 1*.

**Enzyme MBD4^cat^**

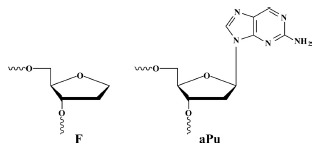



The catalytic domain of human DNA glycosylase, MBD4^cat^ (amino acid residues 426–580), was isolated from the cells of *Escherichia coli* Rosetta 2 transformed with the plasmid pET29b-MBD4cat as described previously [11, 14]. The plasmid pET29b-MBD4^cat^ containing the MBD4^cat^ gene was kindly provided by M.K. Saparbaev (Groupe Réparation de l’ADN, Université Paris-Sud XI, Institut Gustave Roussy, France). The cell culture of *E. coli *Rosetta 2 was grown in a LB medium (1 L) containing 50 μg/mL of kanamycin at 37°C to an optical density of 0.6–0.7 at 600 nm. Then, the temperature was lowered to 20°C and transcription was induced by the addition of isopropyl-β-*D*-thiogalactopyranoside to 0.2 mM. After induction, the cells were incubated during 16 h and then centrifuged (12,000 rpm, 10 min). A cell suspension was prepared in 30 mL of buffer solution I (20 mM HEPES-NaOH, pH 7.8) containing 50 mM KCl. The cells were lysed under pressure using a SIM AMINCO French Press. All subsequent procedures were performed at 4°C. The cell lysate was centrifuged (30,000 rpm, 40 min), and the supernatant was loaded on column I (Q-Sepharose Fast Flow, Amersham Biosciences, Sweden) and eluted with buffer solution I (20 mM HEPES-NaOH, pH 7.8, containing 50 mM KCl). Fractions containing the protein were collected and loaded on column II (HiTrap-Helating™, Amersham Biosciences, Sweden) in buffer solution II (20 mM HEPES-NaOH, pH 7.8, containing 500 mM NaCl and 20 mM imidazole). Chromatography was performed in buffer solution II and a linear gradient of 20 → 500 mM imidazole. The solution’s absorbance was detected at a wavelength of 280 nm. The protein purity was assessed using gel-electrophoresis. Fractions containing the MBD4cat protein were dialyzed in buffer (20 mM HEPES-NaOH, pH 7.5, 1 mM EDTA, 1 mM dithiothreitol, 250 mM NaCl, 50% glycerol) and stored at –20°C. The protein concentration was calculated based on the optical density of the protein solution at 280 nm and a molar extinction coefficient of 54493 M^-1^ cm^-1^ [15].

All the experiments in studying the enzymatic reaction were performed in a buffer solution: 50 mM Tris- HCl, pH 7.5, 50 mM KCl, 1 mM EDTA, 1 mM dithiothreitol, and 9% glycerol at 25°C.

**PAGE product analysis**

The reaction products were separated using polyacrylamide gel electrophoresis (PAGE) with 5’-end 32P-labeled oligonucleotides containing a modified base. The oligonucleotides were labeled at the 5’-end according to [16]. The dependence of the substrate conversion extent on time was studied by mixing 10 μL of a buffer solution containing a 32P-labeled oligonucleotide and an equimolar amount of a complementary oligonucleotide with 10 μL of 2.0–4.0 μM of the enzyme in the same buffer solution. The reaction mixture was rapidly mixed, and after periods of time, 2 μL aliquots were taken and transferred in prepared test tubes containing 2 μL of a 7 M urea solution, 0.1% bromophenol blue, and 0.1% xylene cyanol. Then, 1 μL of 1 M NaOH were added and incubated at 56oC for 15 min to hydrolyse phosphodiester bonds at the AP-sites. The solution was neutralized with an equivalent amount of hydrochloric acid and loaded on a polyacrylamide gel (PAAG). Electrophoresis was performed at a voltage of 50 V/cm. The gel was autoradiographed using the Molecular Imager FX phosphorimager (Bio-Rad, USA), and the data were processed using the Gel-Pro Analyzer 4.0 software package (Media Cybernetics, USA) to determine the amount of the formed product. The degree of product formation was defined as the ratio of the product peak areas to the overall product peak areas and original oligonucleotide peak areas. The measurement error commonly did not exceed 20%.

**Study of the kinetics using the stopped-flow method**

The kinetic curves of fluorescence were detected by a stopped-flow method using an SX.18MV spectrometer (Applied Photophysics, United Kingdom). The MBD4cat protein contains eight Trp residues and seven Tyr residues. Fluorescence excitation of MBD4cat was performed at a wavelength of 290 nm, and the fluorescence was recorded at wavelengths longer than 320 nm (WG-320 filter, Schott, Germany). Under these conditions, Trp residues (> 90%) contributed most to the protein fluorescence. When using substrates containing aPu residues, the fluorescence was excited at wavelengths of 310 nm and detected at wavelengths longer than 370 nm (LG-370 filter, Corion, USA). To analyze the efficiency of FRET energy transfer using the FAM/BHQ1 pair, the fluorescence of the FAM dye was excited at 494 nm and the fluorescence of the FAM dye was detected at wavelengths longer than 515 nm using an OG-515 filter (Schott, Germany). The dead time comprised 1.4 ms. Each kinetic curve was derived by averaging at least three experimental curves.

**Analysis of the kinetic data**

To generate a minimal kinetic scheme that describes the interaction of the enzyme with the substrates and to assess the rate constants for conformational transitions during all the elementary steps of the reaction, a number of kinetic curves for different concentrations of the enzyme or substrate were obtained. The quantitative analysis was performed using the DynaFit software (BioKin, USA) [17] by fitting the parameters to the kinetic schemes as described previously [18-20].

## RESULTS

**Interaction of MBD4^cat^ with the 28-bp DNA-duplex**

**Fig. 2:**
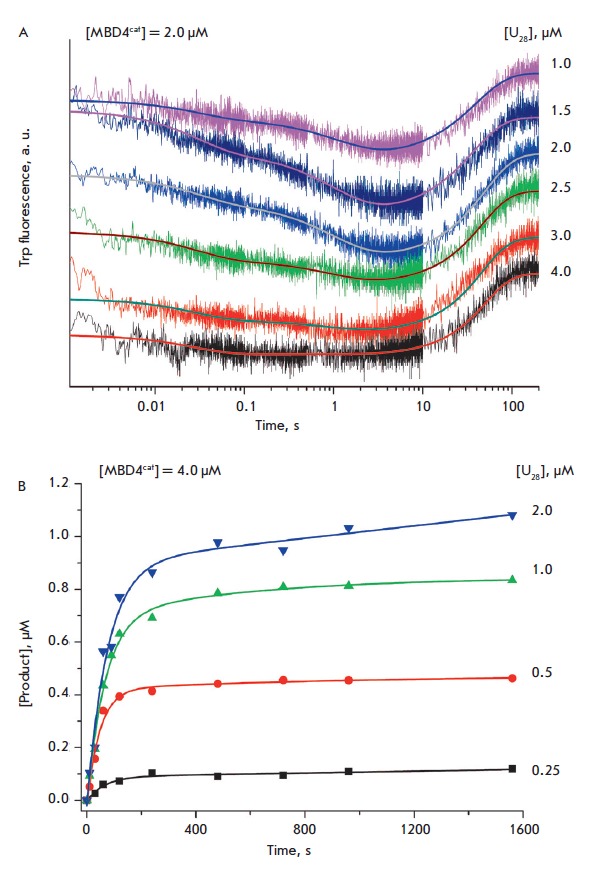
Interaction of MBD4^cat^ with the U_28_-substrate. (A) Changes in the Trp fluorescence intensity. Jagged traces represent the experimental data; smooth curves are the results of the fitting to Scheme 1. [MBD4^cat^] = 2.0 μM, concentrations of U_28_ (1.0–4.0 μM) are shown on the right side of the plot. (B) Accumulation of reaction product determined by PAGE. [MBD4^cat^] = 4.0 μM, concentrations of U_28_ (0.25–2.0 μM) are shown on the right side of the plot.

*Fig. 2A* shows the kinetic curves of the changes in the Trp fluorescence intensity during the interaction of MBD4cat with the 28-bp substrate U_28_. Since MBD4cat forms contacts only with five of the nucleotides of DNA, using the 28-bp DNA duplex as a substrate leads to the fact that the stage of the search for the uracil residue in DNA contributes significantly to the overall rate of the reaction. Indeed, it is seen in the kinetic curves (Fig. 2A) that the fluorescence intensity of Trp decreases within a wide time range 2 ms–10 s followed by a phase of Trp fluorescence intensity growth (10– 100 s).

An analysis of the reaction product formation using electrophoretic separation of the reaction mixture in PAAG (*Fig. 2B*) has shown that the reaction is biphasic, with rapid (to ~200 s) and slow (to 1,600 s and longer) phases. At the initial region of the kinetic curves (to 200 s), a burst is observed, followed by slow growth. This type of curve indicates the presence of a rate-limiting step after the catalytic reaction. An increase in the concentration of U_28_ from 0.25 to 1.0 μM increases the quantity of the cleaved substrate at the step of burst to 0.7 μM. However, with further increase in the concentration of the U_28_-substrate to 2.0 μM, the quantity of the cleaved substrate increases insignificantly to 0.9 μM, indicating that the active form of the enzyme becomes saturated with the DNA substrate. Thus, data on the reaction product formation show that the concentration of the active form of the enzyme is about 1.0 μM. The relatively low activity of the MBD4^cat^ catalytic domain (~ 25%) is apparently linked to the features of the polypeptide assembly into a functional protein in the cells of *E. coli*, which is consistent with the data in [13].

**Scheme 1:**
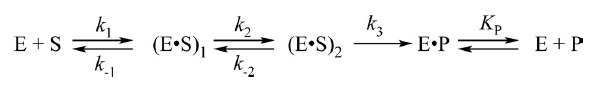
The kinetic mechanism of interaction between MBD4 and a DNA substrate, where E is MBD4; S is the substrate; (E•S)1 and (E•S)2 are enzyme–substrate complexes; (E•P) is a complex of the enzyme with the product; P is the product; *k*i and *k*-i (i = 1 or 2) are the rate constants of equilibrium steps; *k*3 is the rate constant of the catalytic reaction; and *K*p is the equilibrium dissociation constant of the (E•P) complex.

Therefore, it is possible to suggest that the decrease in the Trp fluorescence intensity in the range of 2 ms– ~10 s characterizes the formation of a primary complex and the search for a modified base and growth in an interval of ~10–100 s indicates a reorganization of the enzyme globule into a catalytically competent conformation and onset of product formation. It is necessary to note that an increase in the fluorescence intensity of Trp at an interval of 10–100 s (*Fig. 2A*) coincides in time with the onset of reaction product formation (*Fig. 2B*). The dissociation step of the enzyme–product complex is probably the rate-limiting step of the enzymatic reaction, which is consistent with the conclusions made previously in [10].

Based on a quantitative analysis of the kinetic curves of the MBD4^cat^ interaction with the 28-bp DNA substrate (*Fig. 2A*), we propose the minimal kinetic *Scheme 1* that satisfactorily describes the experimental data. Two equilibrium steps characterize the primary binding with DNA and the subsequent rearrangement of the enzyme conformation, leading to the formation of a catalytically competent complex in which the N-glycoside bond undergoes irreversible hydrolysis. The enzymatic cycle is terminated with the equilibrium dissociation step of the enzyme-DNA product complex. The rate constants and equilibrium constants characterizing the steps contained in *Scheme 1* are presented in *Table 2*. Using the rate constants of elementary steps, steady-state parameter values of the enzymatic reaction were obtained (*K*_m_ and *k*_cat_), which are in good agreement with the data in [11, 13].

**Table 2:** Structures of K_V_-channels alone and in complex with charybdotoxin used in homology modeling studies

Substrates Constants	U_28_	U_17_	U_12_	F_12_
k_1_ × 10^-6^, s^-1^∙M^-1^	7.0 ± 2.0	12.0 ± 3.0	0.5 ± 0.2	10.0 ± 4.0
k^-1^, s^-1^	30 ± 10	45 ± 20	3.3 ± 0.5	8.0 ± 4.0
^a^K_1_ × 10^-6^, M^-1^	0.2 ± 0.1	0.3 ± 0.1	0.15 ± 0.06	1.2 ± 0.8
k_2_, s^-1^	2.1 ± 0.3	3.0 ± 1.0	0.18 ± 0.04	0.08 ± 0.01
k_-2_, s^-1^	0.38 ± 0.05	0.23 ± 0.05	0.12 ± 0.01	0.03 ± 0.01
^a^K_2_	5.5 ± 1.1	13.0 ± 5.0	1.5 ± 0.3	2.7 ± 0.9
k_3_, s^-1^	0.056 ± 0.007	0.046 ± 0.002	< 0.01	
K_P_, M	(1.0 ± 0.2) × 10^-7^	(1.5 ± 0.2) × 10^-7^		
^b^K_d_, M	6.6 × 10^-7^	2.7 × 10^-7^	2.6 × 10^-6^	2.2 × 10_-7_
^c^K_m_, M	7.4 × 10^-7^	3.2 × 10^-7^		
^d^k_cat_, s^-1^	4.6 × 10^-2^	4.2 × 10^-2^		

^a^K_1_ = k_1_/k_-1_, K_2_ = k_2_/k_-2_,

^b^K_d_ = 1/K_ass_, K_ass_ = K_1_ + K_1_ × K_2_,

^c^K_m_ = (k_2_k_3_ + k_-1_k_-2_ + k_-1_k_3_)/k_1_(k_2_ + k_-2_ + k_3_),

^d^k_cat_ = k_2_k_3_/(k_2_ + k_-2_ + k_3_).

Using equation ( 1 ) and the data (*e*_0_ = 4.0 μM, *s*_0_ = 0.5 μM, Fig. 2B), we evaluated the actual rate of steady-state phase of the reaction *V*st ≈ (3.2 ± 1.2) × 10^-5^ μM/s. The expected value of the minimal catalytic reaction rate was estimated with a formula, *V*_max_ = *k*_cat_ × *e*_0_, where *e*_0_ – the initial enzyme concentration: it is 4.6 × 10^-2^ μM/s taking into account 25% enzyme activity. This indicates that the dissociation of the enzyme/reaction product complex, rather than the chemical step, is probably the rate-limiting step, which correlates with the presence of a burst on the kinetic curves of product formation:

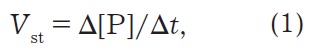



where ∆[P] – an increase in the product P concentration over time ∆*t*.

**Interaction of MBD4cat with the 17-bp DNA-duplex**

**Fig. 3:**
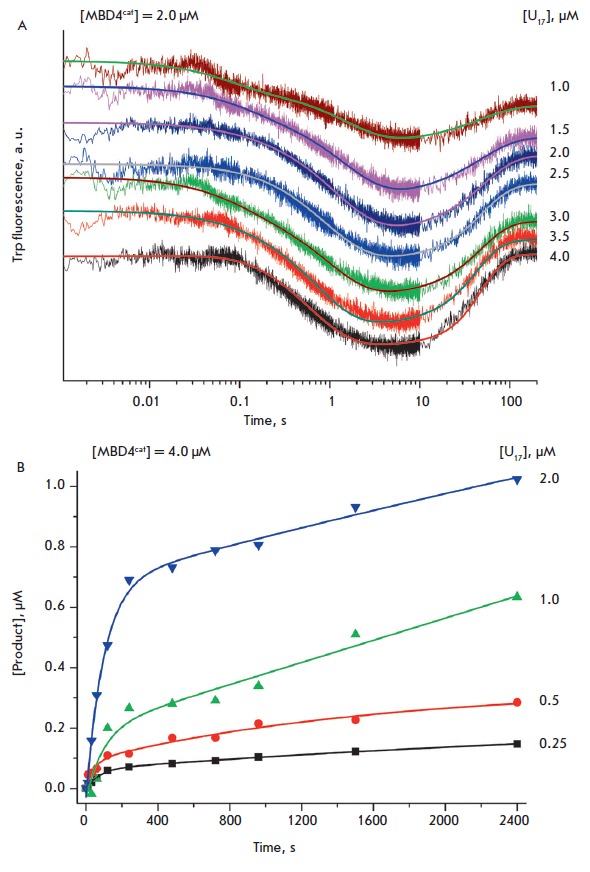
Interaction of MBD4^cat^ with the U_17_-substrate. (A) Changes in the Trp fluorescence intensity. Jagged traces represent experimental data; smooth curves are the results of the data fitting to Scheme 1. [MBD4^cat^] = 2.0 μM, concentrations of U_17_ (1.0–4.0 μM) are shown on the right side of the plot. (B) Accumulation of product determined by PAGE. [MBD4^cat^] = 4.0 μM, concentrations of U_17_ (0.25–2.0 μM) are shown on the right side of the plot.

The kinetic curves obtained during the interaction of MBD4cat with a shorter 17-bp DNA substrate have two phases of changes in the Trp fluorescence intensity, similar to the 28-bp duplex (*Fig. 3A*). An analysis of the reaction products using electrophoretic separation of the reaction mixture in PAAG (*Fig. 3B*) has shown that, in the case of the U17-substrate, the products accumulate within a time interval of < 1,000 s. The kinetic curves have a marked burst of product formation in a time interval of up to 200 s, followed by a slow increase in the product concentration. The subsequent slowdown in the reaction product formation suggests that the dissociation of the enzyme–product complex is the rate-limiting step, similar to the U_28_-substrate.

An analysis of the kinetic curves of fluorescence presented in *Fig. 3A*has shown that the kinetic curves are also described by minimal kinetic *Scheme 1*. The rate and equilibrium constants corresponding to this scheme are presented in *Table 2*. It is notable that the decrease in the non-specific region of the duplex U17-substrate by 11 nucleotides does significantly change the rate constants of formation and decomposition of the (E•S)_1_ primary complex compared to the U_28_-substrate. However, a decrease in the length of the duplex resulted in an increase in the equilibrium constant *K*2, which characterizes the formation of the (E•S)_2_ catalytic complex, 2.4-fold efficiently versus the U_28_-substrate (13.0 and 5.5, respectively). This should be associated with a reduction in the search time for modified bases, which occurs through one-dimensional diffusion by means of both sliding and hopping [21-23] of the enzyme along the DNA chain and three-dimensional diffusion that involves multiple acts of association-dissociation.

Similar to the case of the U_28_-substrate, the U_17_-substrate is characterized by an actual steady-state rate of product formation *V*st of ~ (6.2 ± 0.9) × 10-5 μM/s at *e*0 = 4.0 μM, *s*0 = 0.5 μM and a lower value *V*max = 4.2 × 10^-2^ almost 1,000-fold. This difference, as well as the burst on the kinetic curves of reaction product formation (*Fig. 3B*), indicates that the dissociation of the enzyme-reaction product complex limits the rate of the enzymatic reaction.

**Interaction of MBD4cat with the 12-bp DNA-duplex**

**Fig. 4:**
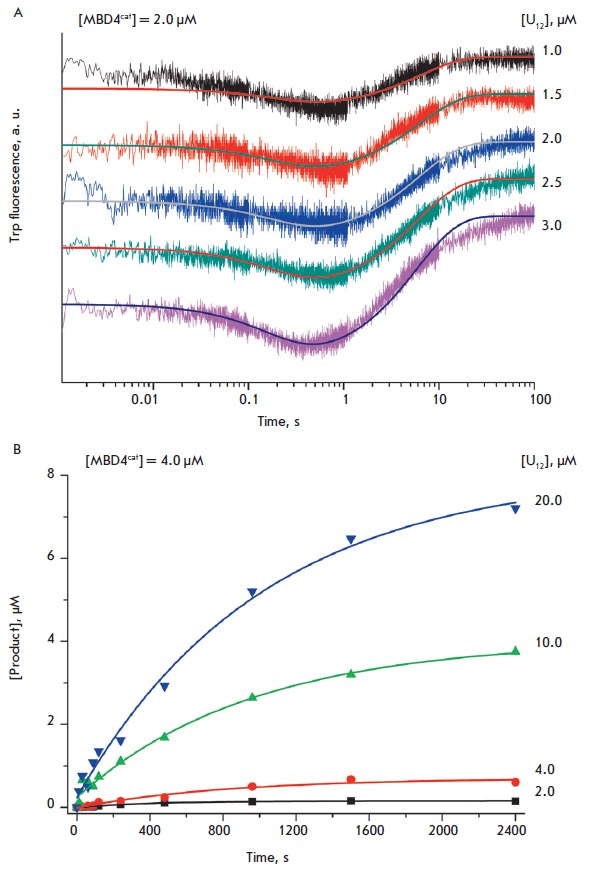
Interaction of MBD4^cat^ with U_12_-substrate. (A) Changes in the Trp fluorescence intensity. Jagged traces represent experimental data; smooth curves are the results of the data fitting to Scheme 1 (steps 1 and 2). [MBD4^cat^] = 2.0 μM, concentrations of U_12_ (1.0–3.0 μM) are shown on the right side of the plot. (B) Accumulation of product determined by PAGE. [MBD4^cat^] = 4.0 μM, concentrations of U_12_ (2.0–20.0 μM) are shown on the right side of the plot.

*Fig. 4* shows the curves of the changes in the Trp fluorescence intensity corresponding to the interaction of MBD4cat with the 12-bp substrate U12. *Fig. 4A* reveals that the kinetic curves have two phases: a decrease in the intensity (10–500 ms) and growth reaching a plateau (0.5–10 s). The kinetics of product formation determined by electrophoretic separation of the reaction mixture in PAAG (*Fig. 4B*) suggests that the catalytic phase occurs at time points ≥ 1,000 s, i.e., much slower than the steps corresponding to the change in the Trp fluorescence intensity. Therefore, these changes that are detected in the time interval to 200 s most likely reflect the initial conformational changes in the enzyme during enzyme-substrate complex formation and further rearrangement in the enzyme conformation. In addition, a catalytically competent state is formed with poor efficiency, and the reaction of N-glycoside bond hydrolysis is significantly slowed down compared with the 17- and 28-bp substrates. Therefore, to analyze a series of kinetic curves, we used only the equilibrium steps of DNA binding that characterize the formation of the (E•S)_1_ and (E•S)_2_ complexes on *Scheme 1*. The rate constants characterizing these steps and derived by analyzing the kinetic curves of fluorescence intensity changes of the enzyme (*Fig. 4A*) are presented in *Table 2*.

A comparison of the rate constants corresponding to MBD4^cat^ interaction with the 12- and 17-bp substrates reveals that a decrease in the duplex length by five nucleotides reduced significantly both the rate of the (E•S)_1_ primary complex formation, which is characterized by the rate constant *k*_1_, and the rate of complex decomposition, *k-*_1_, 24- and 14-fold, respectively. Furthermore, the association constant of E with S, resulting in the (E•S)_1_ complex formation, *K*_1_, decreases only 2-fold. It is suggested that the nonspecific region of the U_17_-substrate is a template with which the primary complex (E•S)_1_ is formed under conditions of rapid equilibrium that allows the enzyme to perform an effective search for a damaged site. In fact, the constant *k*_2_ of formation of the catalytic complex (E•S)_2_ in the case of the U_17_-substrate is 17-fold higher than in the case of the U_12_-substrate. Thus, an increase in the nonspecific region of the duplex by five nucleotides in the U_17_-substrate compared to the U_12_-substrate results in a 10-fold decrease in the total dissociation constant *K*d (*Table 2*). Absence of a nonspecific region in the U_12_-substrate, apparently, prevents the formation of a properly oriented active conformation of the enzyme.

**Interaction of MBD4cat with an analogue of the product**

Excision of the modified base by the MBD4 enzyme leads to the formation of an AP-site in DNA. In order to identify the nature of the conformational changes in the enzyme and DNA that occur during binding of the reaction product containing the AP-site, we used a stable AP-site analogue, a (3-hydroxytetrahydrofuran-2-yl) methyl phosphate residue (F-site) without an OH-group in the C1’ position of deoxyribose. An interaction of MBD4^cat^ with the F12-ligand should lead to the formation of a complex that imitates the enzyme/product complex after the catalytic steps of the enzymatic reaction.

**Fig. 5:**
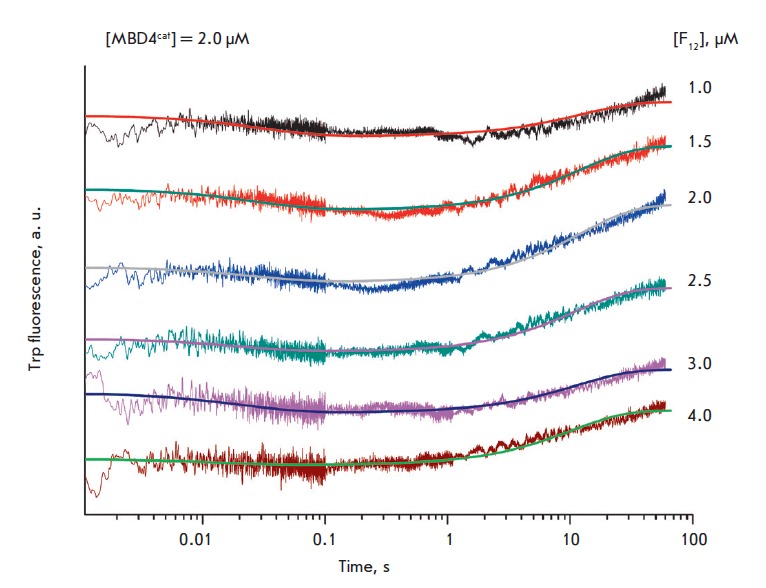
Changes in the Trp fluorescence intensity during the interaction of MBD4^cat^ with F_12_-ligand. Jagged traces represent experimental data; smooth curves are the results of the fitting to Scheme 1 (steps 1 and 2). [MBD4^cat^] = 2.0 μM, concentrations of F_12_ (1.0–4.0 μM) are shown on the right side of the plot.

The kinetic curves of the changes in the Trp fluorescence intensity during the interaction of MBD4^cat^ and the F_12_-ligand have a biphasic profile (*Fig. 5*), similar to the U_12_-substrate (*Fig. 4*). It can be suggested that the decrease and subsequent increase in the Trp fluorescence intensity have the same nature as in the DNA duplex containing uridine. Thus, regardless of the nature of the modified nucleotide, primary binding and further rearrangement of the enzyme conformation during the interaction of MBD4cat with DNA lead to the formation of a catalytically active complex (*Fig. 5*). The derived kinetic curves are described satisfactorily by a two-step equilibrium kinetic scheme. The rate constants characterizing these steps are presented in *Table 2*.

A comparison of the rate constants and equilibrium constants (* Table 2 *) characterizing the interaction of MBD4cat with the U12-substrate and F12-ligand has shown that a primary complex with a reaction product analogue is formed with about a 10-fold higher efficiency. It is noteworthy that the equilibrium constants for the second step differ less than 2-fold. Hence, we can conclude that the search for and binding of damaged DNA by the MBD4cat enzyme occur with higher efficiency in the case of a DNA duplex destabilized by an F-site.

**Comparative analysis of conformational changes in the enzyme and DNA**

**Fig. 6:**
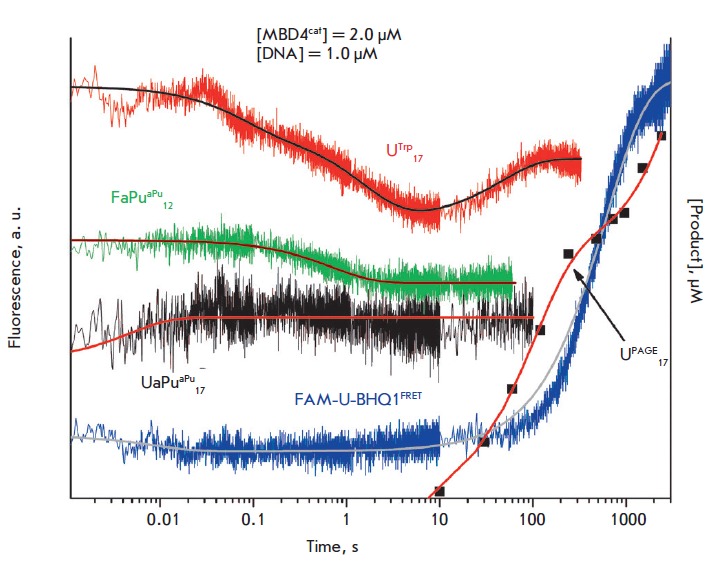
PAGE and changes in the fluorescence intensity of the Trp and aPu residues and the FRET-signal during the interaction of MBD4^cat^ with U_17_, UaPu_17_, FaPu_12_ and FAM-U-BHQ1. [MBD4^cat^] = 2.0 μM, [DNA] = 1.0 μM.

In order to clarify the nature of the processes occurring during catalytic complex formation and detected from the changes in the Trp fluorescence intensity, we used model DNA duplexes carrying fluorescent residues (Table 1) as “sensors” of the conformational changes in DNA. It is known that the fluorescence intensity of aPu in DNA depends on the fluorophore microenvironment changes, for example, as a result of local melting of the duplex in the immediate vicinity of the fluorophore [24-26]. In double-stranded DNA structures, during stacking interaction with neighboring bases, the fluorescence intensity of aPu residues reduces significantly in comparison with single-stranded DNA. Using a UaPu_17_-substrate carrying a aPu residue on the 3’-side of uridine allowed us to detect the conformational changes in DNA that occur during the interaction with MBD4^cat^, probably, as a result of an eversion of the damaged nucleotide from DNA and insertion of the Arg468 and Leu508 residues into the resulting void. As seen in *Fig. 6*, the kinetic curve which characterizes the interaction of the MBD4cat and UaPu_17_-substrate has a phase of rapid growth (1–50 ms) of the aPu fluorescence intensity, indicating a destabilization of the central part of the duplex at the initial steps of enzyme-substrate interaction.

Additionally, a FRET technique was applied to study changes in the DNA-substrate structure. We used a FAM-U-BHQ1-substrate containing FAM and BHQ1 dyes at the 5′-ends of the oligonucleotides forming the duplex to track the conformational changes in the duplex linked to the changes in the distance between the donor and acceptor dye molecules. It is clear (*Fig. 6*) that a small decrease in the FRET-signal occurs at the initial region of the kinetic curve (1–50 ms) indicating a decrease in the distance between FAM and BHQ1 in the duplex. A comparison with a curve of the changes in the Trp fluorescence intensity has shown that at this time point ( < 100 ms), the enzyme undergoes no significant conformational changes. Therefore, we can suggest that during the initial DNA binding the helix is bent and the central part of the duplex partially melts. Apparently, it is exactly these changes in the structure of the duplex induced by the enzyme that allow MBD4cat to distinguish between the modified and non-modified bases during the sliding on the DNA duplex.

Interestingly, the growth phase of the Trp fluorescence intensity (10–100 s), coincident with the initial reaction product formation, does not correspond with the change in the FRET signal. However, the rate-limiting step of the enzyme/reaction product complex dissociation causes a slow increase in the FRET signal in a time interval of 100–3,000 s and also leads to a slow accumulation of reaction products (*Fig. 6*).

The intensity of the aPu fluorescence upon binding with DNA-duplexes containing uracil (UaPu_17_) and the F-site (FaPu_12_) changes oppositely (an increase and a decrease, respectively) and in different time intervals (*t* < 0.1 s and 0.1 s < *t* < 1 s, respectively). The growth phase of the aPu fluorescence intensity when using the UaPu_17_-substrate reflects a local melting of the duplex during primary complex formation. While this growth phase on the aPu fluorescence traces are not detected when using a FaPu_12_-ligand, since the aPu residue located on the 3′-side from the F-site is already situated in a less hydrophobic environment than in canonical DNA. However, during the interaction of MBD4^cat^ with the FaPu_12_-ligand, a well-marked decrease in the aPu fluorescence intensity is observed at later time points (up to 1 s). This decrease, associated with an increased hydrophobicity of the aPu residue environment, most likely indicates an insertion of the amino acid residues of MBD4^cat^ (Arg468 and Leu508) into the duplex at these time points.

## DISCUSSION

X-ray crystallography findings [11, 12] allow one to suggest that in the primary complex, formed during the interaction of MBD4cat with DNA in the initial time point, amino acid residues build a system of non-specific contacts with the ribose-phosphate backbone, allowing the enzyme, due to thermal motions, to move along the double helix in search of a damaged nucleotide. It is known that the search for specific sites by enzymes in a DNA molecule occurs by using both one-dimensional (1D) and three-dimensional (3D) diffusion: without dissociation of the enzyme-substrate complex by means of enzyme sliding and hopping along the DNA chain and by multiple acts of dissociation-association, respectively [21, 23]. The length of the 1D-diffusion region differs for different DNA-glycosylases. It was shown that uracil-DNA-glycosylase UNG can move by a length of about four nucleotides between acts of dissociation [27]. In 8-oxoguanine-DNA-glycosylase hOGG1, this distance may range from 60 [28] to 400 [29] nucleotides. DNA glycosylases Fpg, Nei, Nth, and hOGG1 [30-32] use certain residues as detectors for a damaged region of DNA during the search for a damaged site in the DNA molecule. If a modified nucleotide appears in the DNA-binding center of the enzyme, this leads to the formation of specific contacts, an eversion of the nucleotide from the DNA duplex, the insertion of a modified base into the active site of the enzyme, and movement of detector amino acid residues into the void in the DNA duplex. These conformational rearrangements prevent the enzyme from moving from the specific site on the DNA and lead to the formation of a catalytically competent complex. It is interesting to note that the MBD4cat enzyme, which belongs to the structural family of a HhH motif containing DNA-glycosylases together with hOGG1 and Nth, also inserts Arg468 and Leu508 into the DNA duplex during catalytic complex formation. Therefore, these amino acid residues can function as detectors during the search for a damaged nucleotide.

Comparison of fluorescence kinetics curves for the 12-, 17-, and 28-bp DNA substrates shows that with increasing duplex length, the phase of decrease in the Trp fluorescence intensity slows down, which characterizes the search for the modified base during sliding or hopping of MBD4^cat^ on the DNA chain. Using fluorescently labeled UaPu_17_- and FAM-U-BHQ1-substrates, it has been shown that the DNA duplex bends and locally melts in the primary complex. In the subsequent time point, the damaged nucleotide is everted from the duplex and the amino acid residues of the enzyme are inserted in the void. The results obtained with a fluorescently labeled analogue of the reaction product FaPu_12_- show that insertion of Arg468 and Leu508 in the DNA duplex is preceded by the growth phase of Trp fluorescence intensity. Insertion of amino acid residues into the DNA provides for specific recognition of the modified base. The growth phase of Trp fluorescence intensity characterizes the formation of a catalytically active complex and ends in 100–200 s in all cases, which coincides with the initial burst of reaction product formation. Dissociation of the reaction product/enzyme complex is the rate-limiting step of the entire reaction. The observed rate of the catalytic reaction increases markedly with increased length of the duplex. This indicates the possible involvement of the non-modified DNA regions lying adjacent to the modified base in the formation of a properly oriented and active conformation of MBD4^cat^.

Thus, conformational transitions in the MBD4 enzyme and DNA substrates during their interaction have been detected in real time for the first time. The kinetic mechanism has been established. The rate constants of formation and dissociation of intermediate enzyme-substrate complexes have been calculated, and the nature of the conformational rearrangements of the MBD4cat and DNA substrates during modified base recognition and excision has been identified.

## Abbreviations

MBD4cat: catalytic domain of methyl-CpG-binding enzyme MBD4
5hmU: 5-hydroxymethyluracil
AP-site: apurinic/apyrimidinic site
F-site: (3-hydroxytetrahydrofuran-2-yl) methyl phosphate
aPu: 2-aminopurine
FAM: 6-carboxyfluorescein
BHQ1: black hole quencher
FRET: Förster resonance energy transfer
PAGE: polyacrylamide gel electrophoresis
